# Development, roll-out and implementation of an antimicrobial resistance training curriculum harmonizes delivery of in-service training to healthcare workers in Kenya

**DOI:** 10.3389/fmicb.2023.1142622

**Published:** 2023-08-01

**Authors:** Josiah Njeru, Joshua Odero, Sheilla Chebore, Mungai Ndung’u, Emmanuel Tanui, Evelyn Wesangula, Romona Ndanyi, Susan Githii, Revathi Gunturu, Willy Mwangi, David Mutonga, Anicet Dahourou, Andrew Thaiyah

**Affiliations:** ^1^USAID Infectious Disease Detection and Surveillance (IDDS) Project, Nairobi, Kenya; ^2^National Antimicrobial Stewardship Interagency Committee (NASIC), Ministry of Health, Nairobi, Kenya; ^3^Directorate of Patient and Health Workers Safety, Ministry of Health, Nairobi, Kenya; ^4^Department of Veterinary Services, Ministry of Agriculture, Livestock, Fisheries and Cooperatives, Nairobi, Kenya; ^5^National Public Health Laboratories, Ministry of Health, Nairobi, Kenya; ^6^Aga Khan University Hospital, Nairobi, Kenya; ^7^University of Nairobi, Nairobi, Kenya; ^8^USAID Infectious Disease Detection and Surveillance (IDDS) Project, Fairfax, VA, United States; ^9^USAID Mission in Kenya and East Africa, Nairobi, Kenya

**Keywords:** antimicrobial resistance, One Health, bacteriology, healthcare workforce, Kenya, training curriculum, in-service training

## Abstract

**Background:**

Antimicrobial resistance (AMR) is an increasingly severe threat to global public health that requires action across different sectors. Selection of appropriate antimicrobials is an urgent challenge due to the emergence of drug resistance. In 2017, Kenya developed an AMR policy and National Action Plan to drive prevention and containment of AMR. A priority activity under AMR surveillance strategic objective was to develop a national AMR training curriculum for in-service healthcare workers. In this paper we discuss the development process, gains achieved through implementation across the country and lessons learned.

**Methods:**

An initial stakeholders’ forum was convened to brainstorm on the process for developing the curriculum and some issues deliberated upon include the design approach, development roadmap, curriculum outline and scope, delivery, and evaluation methodologies. A dedicated team of subject matter experts (SMEs), drawn from the project and government ministries, compiled the initial draft of the curriculum and later the training materials. A series of other stakeholders’ meetings were convened to review these materials. The National Antimicrobial Stewardship Interagency Committee (NASIC) of the MOH in Kenya identified a team of experts from academia, research, and government to work with the SMEs in reviewing and providing valuable inputs to the curriculum. Additionally, principles of adult learning and a One Health approach for development were considered as AMR has drivers and impacts across sectors. A validation workshop was held to finalize the documents with a formal launch conducted during the World Antibiotics Awareness Week of 2020.

**Results:**

A multisectoral AMR surveillance training curriculum and facilitator and trainee manuals were developed and endorsed by MOH and Ministry of Agriculture, Livestock, Fisheries and Cooperatives within one year. Over 500 healthcare workers in 19 counties were trained, with overwhelming adoption by other stakeholders in Kenya and beyond.

**Conclusion:**

This curriculum was developed to standardize training for AMR detection and surveillance. The central role played by the MOH ensured expeditious development and roll-out of this curriculum. The in-service curriculum, now available on an e-learning platform, provides a ready opportunity to build capacity of healthcare professionals. Additional resources are needed to standardize and scale these efforts to reach all healthcare workers.

## Introduction

Antimicrobial resistance (AMR) is a growing global public health threat requiring concerted efforts across human, animal, and environmental health sectors (Interagency Coordination Group on Antimicrobial Resistance [IACG], 2019; Pokharel et al., 2020). AMR occurs when microorganisms develop resistance to antimicrobial drugs to which they were previously sensitive. Misuse of antimicrobials in both human and animals is the primary driver of emergence and spread of AMR (Pokharel et al., 2020). Globally, it is projected that by 2050, AMR will result in 10 million human fatalities per year and a 2–3.5 percent decrease in global gross domestic product (Jee et al., 2018; Interagency Coordination Group on Antimicrobial Resistance [IACG], 2019). Several studies and reports in Kenya have documented the increasing trend of antimicrobial use and resistance in both humans and animals. However, the actual trend and exact burden in Kenya is unknown due to lack of systematic surveillance (Global Antibiotic Resistance Partnership—Kenya Working Group, 2011).

In recognition of the growing threat of AMR, in 2015 the World Health Organization developed a Global Action Plan on AMR following a 2014 resolution on AMR at the World Health Assembly (WHA 67.25), which called on the agency to develop a draft global action plan to ensure that all countries have the capacity to combat AMR (World Health Organization, 2015). In 2017, the Kenya ministries responsible for health and agriculture spearheaded the development of a One Health National Policy and Action Plan to combat AMR, in line with the Global Action Plan and International Health Regulations (2005); (Government of Kenya, 2017). Subsequently, a National Antimicrobial Stewardship and Interagency Committee (NASIC) to coordinate efforts across the health and agriculture sectors and other key partners was established. NASIC is multisectoral and interdisciplinary, drawing membership from government, academia, industry, and private sectors, as well as experts in animal, human, and environmental health. NASIC employed a One Health approach in the fight against AMR, recognizing use and misuse of antimicrobials across human, animal, and environmental sectors as drivers of AMR. Resistant bacteria and resistance determinants also spread within and between these sectors around the globe (McEwen and Collignon, 2018).

AMR surveillance is one of the strategic objectives of the Kenya National Action Plan. Other objectives include the need to increase knowledge and awareness of AMR, antimicrobial stewardship, infection prevention and control, and research and development. The Joint External Evaluation conducted in 2017 using the International Health Regulations (2005) benchmarks revealed that Kenya had made progress in strengthening its capacity to prevent, detect, report, and respond to public health threats, but significant gaps remained under the AMR core capacity. AMR detection, surveillance and antimicrobial stewardship indicators each had a low score of two (2) out of the possible score of five (5) (World Health Organization, 2005, 2017). AMR surveillance activities in the country were initiated in 2018 at two human health pilot surveillance sites, with a goal to increase the number of engaged sites gradually (Ministry of Health Kenya, 2018).

The second strategic objective in the Kenya National Action Plan is to strengthen the awareness and the evidence base for AMR (Ministry of Health Kenya, 2017). Knowledge and skills development, for both pre– and in-service health workers, are critical to effectively manage medicines, implement infection prevention and control, and support surveillance activities (Systems for Improved Access to Pharmaceuticals and Services, 2013). An in-service training curriculum allowed the Ministry of Health (MOH) to influence practices among healthcare workers compared to pre-service curricula, which are highly regulated by various government agencies (Ministry of Health Kenya, 2016). In the absence of an in-service standardized training course to guide AMR surveillance in Kenya, NASIC prioritized the development of a standard training course on AMR surveillance to guide training of national and county teams.

AMR surveillance in Kenya, like in many other developing countries, is still in its nascent stages, with limited coverage (Iskandar et al., 2021). The development of this curriculum provides a timely and relevant tool to guide systematic strengthening of knowledge and skills development among in-service healthcare professionals, including those in leadership and governance in both the human and animal health sectors. It also aims to raise awareness and recognition of AMR as a growing public health threat nationally and globally. Finally, improved knowledge of diagnostics and stewardship for AMR would generate evidence for estimation of the exact burden of AMR in the country.

USAID’s Infectious Disease Detection and Surveillance (IDDS) project is a global health security project strengthening infectious diseases detection and surveillance systems in more than 20 countries, including Kenya. In Kenya, the project works closely with NASIC within the MOH to combat AMR nationally. In this article we discuss the technical assistance provided to the Government of Kenya in the development of an in-service AMR surveillance curriculum and training materials, roll-out, and implementation.

## Methods

### AMR learning needs assessment

The need for an in-service AMR surveillance training curriculum was recognized by stakeholders prior to the start of concerted efforts to combat AMR in the country in 2017 (Global Antibiotic Resistance Partnership—Kenya Working Group, 2011). This was later captured as a national priority in the National Action Plan on prevention and containment of AMR in the country in 2017 (Ministry of Health Kenya, 2017). Influencing knowledge, attitudes, and practice among in-service healthcare workers in Kenya was identified as a key opportunity compared to introducing AMR surveillance into pre-service curricula. NASIC continues to engage with academic institutions to increase content for pre-service students on antimicrobial stewardship and AMR as a global public health challenge.

### Design process and the One Health approach

**Figure 1:**
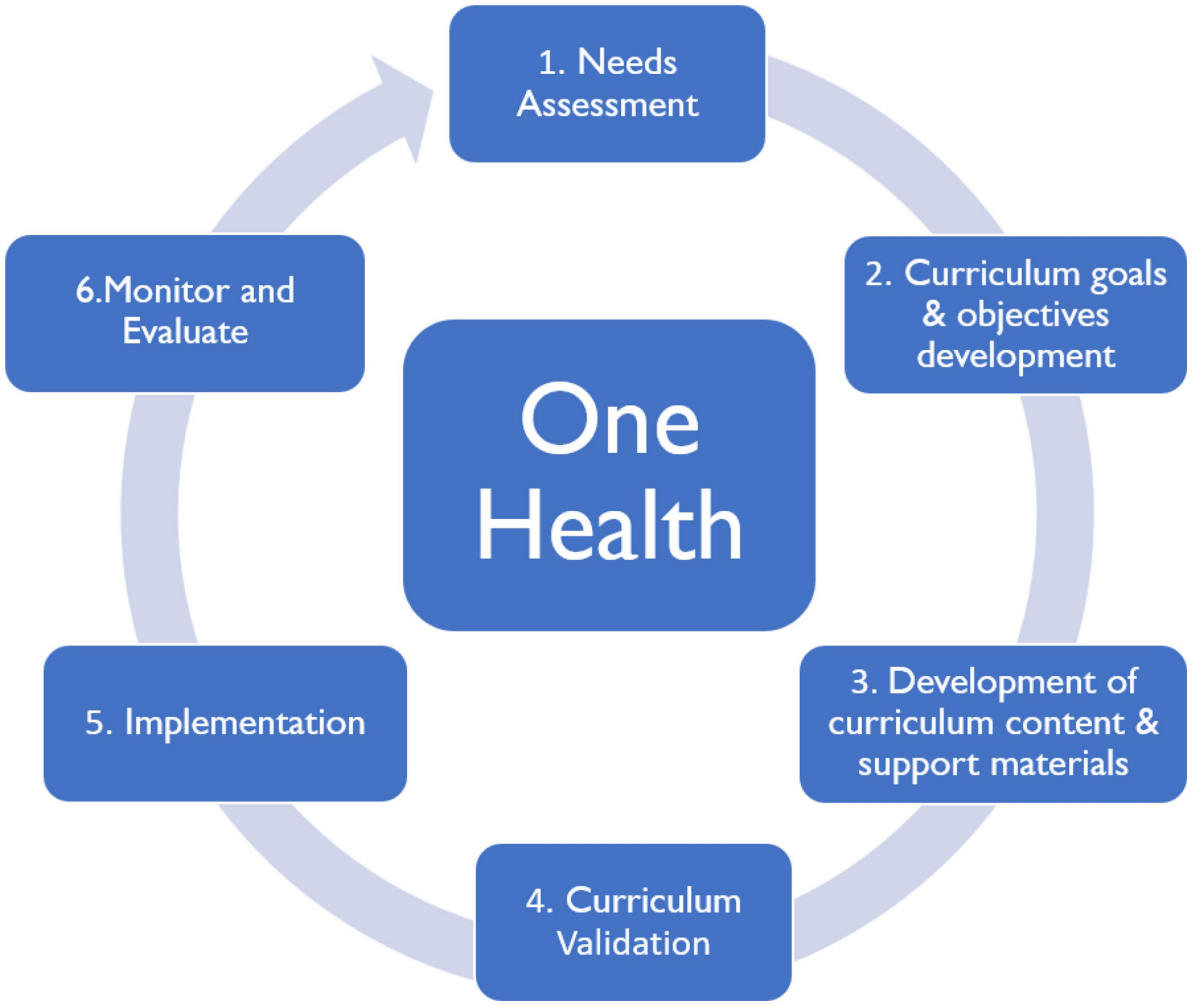
Curriculum design process adopted by stakeholders.

A stakeholders’ forum was convened by NASIC with the support of the USAID IDDS project. An expert from academia made presentations on the curriculum development process. Stakeholders brainstormed several aspects including the curriculum outline and scope, delivery, and evaluation methodologies, and agreed that the new materials would not replace pre-service curricula. Stakeholders emphasized the need to adopt a One Health approach in the development process because AMR drivers are interdisciplinary (Figure 1).

### Development roadmap and milestones

**Figure 2:**
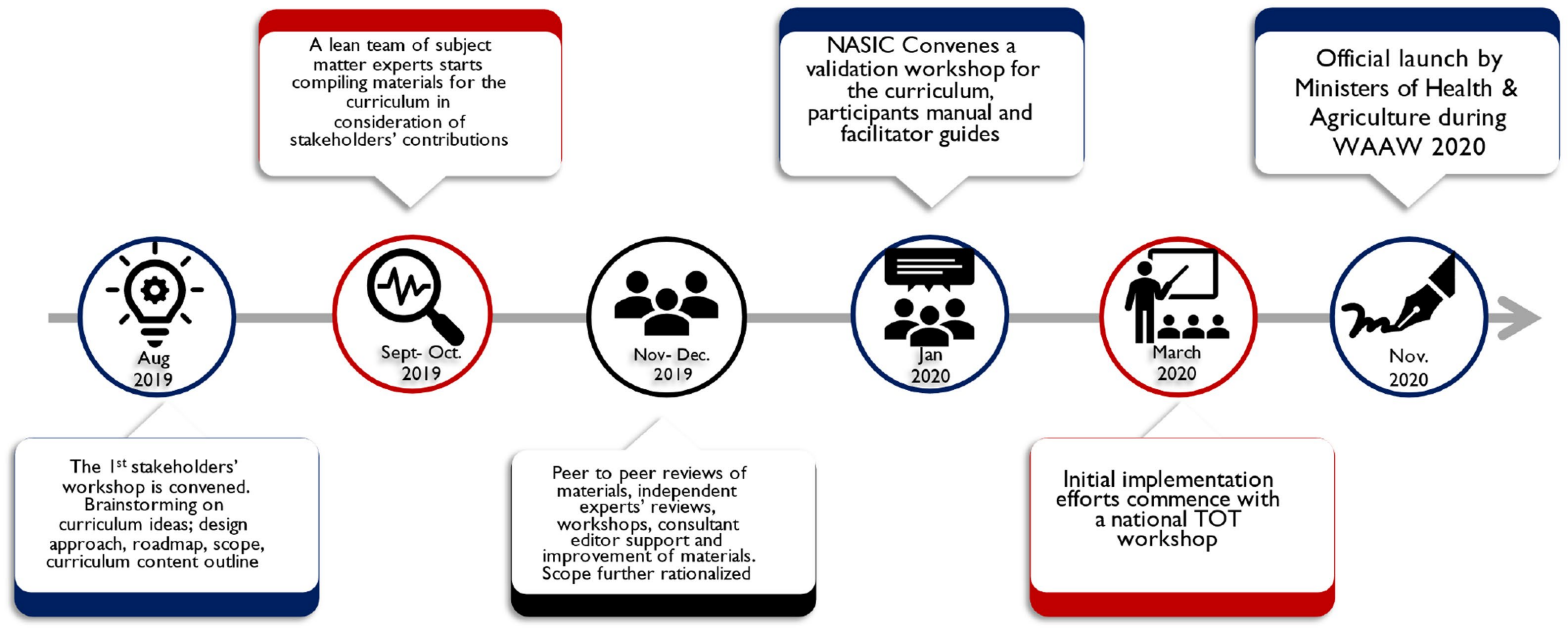
Roadmap of curriculum development.

NASIC convened all stakeholders’ meetings during the development process for review of materials with technical assistance from IDDS. A team of subject matter experts (SMEs) from IDDS, MOH, Ministry of Agriculture, Livestock, Fisheries and Cooperatives, and key academic institutions worked on curriculum outlines, content outlines, intended learner outcomes, training content, evaluations, and a Monitoring & Evaluation plan for the curriculum. These materials were built on the recommendations from the initial stakeholders’ workshop. The team developed participant and facilitator manuals. The SMEs adopted a peer-to-peer review approach where one peer reviewed materials developed by another. The materials were submitted to an in-country panel of experts identified by NASIC. After their reviews, the materials were submitted to external reviewers and editors. A validation workshop was convened for stakeholders to confirm that the curriculum and its related materials captured the national aspiration to fill knowledge and practice gaps among in-service healthcare professionals. Later in the year, the documents were officially launched by MOH and Ministry of Agriculture, Livestock, Fisheries and Cooperatives. Figure 2 provides a summary of the development roadmap.

## Results

### Summary of the Kenya AMR surveillance training curriculum

#### Course objectives

The curriculum was designed to have objectives at two levels: for participants completing pre-analytic modules and participants completing analytical and post-analytic modules. The pre-analytic modules aim to equip participants with knowledge and skills to:

i) Develop and deploy customized antimicrobial use and AMR containment and prevention interventions;
ii) Apply a One Health approach in addressing AMR agenda; and
iii) Coordinate, monitor, and evaluate multisectoral stewardship activities targeted at mitigating AMR.

The analytic and post-analytic modules of the course were designed to equip learners with knowledge and skills to:

i) Utilize advances in diagnostic techniques (e.g., rapid molecular tests, biomarkers) and design methods to incorporate their use/application to stewardship initiatives;
ii) Perform AMR surveillance monitoring, evaluation, and reporting; and
iii) Use antimicrobials judiciously to minimize or eliminate the occurrence of AMR.

#### Course duration

The recommended course duration for laboratory practitioners is five days, due to the hands-on microbiology techniques module, while three days are recommended for other healthcare cadres. This modular approach can be adapted to respond to specific needs of the target audience. Table 1 provides details of the 12 modules and estimated duration for each.

**Table 1:** Summary of the Kenya AMR surveillance curriculum modules.

Module	Module summary	Number of Sub-modules	Estimated module time (hrs)
Module 1: Overview of AMR	Gives a global and national overview of AMR and a summary of strategies Kenya is taking to combat AMR	6	4
Module 2: Drivers of Antimicrobial Resistance at the Human-Animal-Environment Interface	Introduces participants to AMR occurrence at the human-animal-environment interface and the need for a One Health approach	1	0.75
Module 3: Biosafety and Biosecurity	Module gives an overview of biosafety and biosecurity as relates to AMR diagnostics	1	2.5
Module 4: Specimen Collection, Transport, Reception, and Storage	Addresses pre-analytical quality requirements for AMR surveillance specimen, i.e., processes related to specimen collection, transportation to the laboratory, reception, and storage	5	3
Module 5: Microbiological Procedures	Focuses on the laboratory diagnostic processes for organism identification, antimicrobial susceptibility testing, and results reporting	7	>1 day
Module 6: Specimen Referral and Reporting	Covers the role of specimen referral systems in increasing access to diagnostics	1	1.25
Module 7: Quality Assurance in the Clinical Microbiology Laboratory	Covers quality assurance procedures in a microbiology laboratory	1	2.5
Module 8: AMR Surveillance Monitoring, Evaluation, and Reporting	Focuses on all aspects of AMR surveillance data management monitoring, evaluation, and reporting	5	9
Module 9: Procurement and Supply Chain Management	Covers basic concepts of supply chain management and application to AMR surveillance supplies	1	0.75
Module 10: Equipment Management	Covers introduction to equipment management processes	1	2
Module 11: Clinical Guide	Covers approaches to promote prudent use of antimicrobials in human health	1	3.5
Module 12: Prudent Use of Antimicrobials in Veterinary Practice	Covers approaches to promote prudent use of antimicrobials in animal health	2	6.5

#### Target audience

The curriculum mainly targets in-service healthcare workers who play various roles in AMR diagnostics and surveillance across animal and human health sectors. These include clinicians, veterinarians, veterinary paraprofessionals, laboratory personnel, pharmacists, nurses, biomedical engineers, informatics, and records officers. Preliminary modules are designed to raise awareness of AMR, and thus can be offered to leaders for AMR resource mobilization and allocation, especially at the national and county levels.

#### Course organization and delivery

Mixed delivery methods were proposed based on principles of adult learning (McGrath, 2009). Delivery methods vary from one module to another, depending on intended learner outcomes. The methods include lectures with PowerPoint presentations, role plays, demonstration of technical procedures, hands-on practical sessions, trainer-moderated group work sessions, case studies, and use of visual aids.

The curriculum is organized into modules, each focusing on a specific area based on: (a) the need to share information on AMR, or (b) the need to transfer knowledge and skills on AMR diagnosis and surveillance. Table 1 provides an overview of each of the modules, sub-modules, and expected delivery time.

#### Course evaluation and curriculum monitoring and evaluation plan

All modules have multiple choice questions to check participants’ knowledge before and after the training. Both trainer and participant course evaluation forms were developed to capture valuable feedback for improvement of the curriculum and materials. Data collected through these evaluations and other informal feedback mechanisms would be used to assess effectiveness, impact, and inform improvements to the curriculum and course content during the first review cycle (two years after the launch).

#### Curriculum validation

A validation workshop was convened on January 8, 2020, and attended by 63 AMR stakeholders from multiple sectors; these national and sub-national stakeholders reviewed and approved the curriculum and its associated training materials for publication and implementation.

### Implementation

#### National training of trainers

After finalization of the curriculum and training materials, IDDS provided support to NASIC to organize a five-day “training of trainers” session covering the 12 curriculum modules. The training convened participants from human and animal sectors to support roll-out of the curriculum. These included 18 laboratory practitioners, 3 nurses, 3 monitoring and evaluation professionals, 2 pharmacists, 1 public health officer, 1 medical officer, and 2 veterinary officers. Scores increased from a pre-test average of 61% to a post-test average of 87%. This training also served as a pilot through which valuable inputs were provided by both trainers and participants that were helpful with improving the materials. Key feedback guided revision of content scope against time allocated. Some modules content, such as biosafety and biosecurity, were reduced to keep the focus on AMR while more time was allocated to other modules, including the clinical guide.

#### Sensitization of healthcare workers at the county level

In 2020, IDDS, in collaboration with five counties and NASIC, conducted a two-day AMR surveillance sensitization training based on the new curriculum, to create awareness among different stakeholders of AMR and ensure a robust response to the threat of AMR. The objectives of the training were to improve health workers’ awareness and understanding of AMR surveillance in the counties, build capacity on best practices to adopt and promote appropriate use of antibiotics and avert AMR, and how to conduct AMR surveillance in the counties aligned with Kenya policy guidelines on AMR surveillance.

The target audience for the training were clinicians, veterinarians, veterinary paraprofessionals, laboratory personnel, pharmacists, nurses, public health officers, records officers, and health policy– and decision-making authorities.

**Figure 3:**
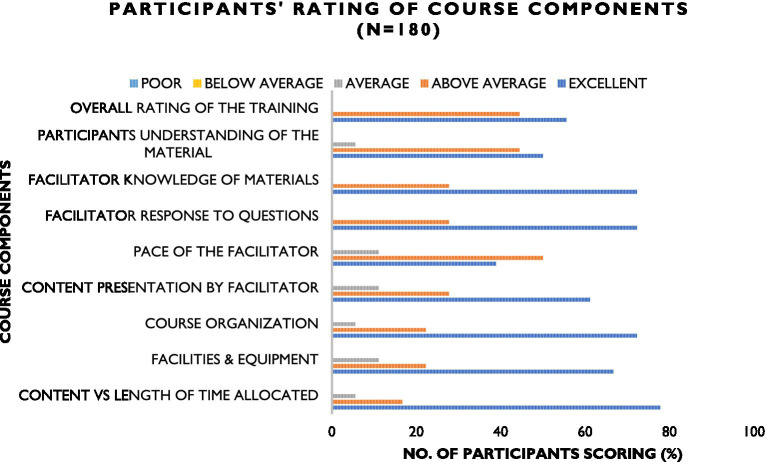
Participants’ rating of the AMR surveillance curriculum content offered during sensitization of clinicians across the 5 supported counties.

A total of nine of the 12 modules in the AMR surveillance training curriculum were delivered. These included foundational modules that built awareness of AMR, appropriate use of antimicrobial agents; infection prevention and control; drivers of AMR at the human-animal-environment interface; diagnostic stewardship; specimen collection, transport, reception, and storage; specimen referral and reporting; procurement and supply chain management; clinical guide; and prudent use of antimicrobials in veterinary practice. These sessions were facilitated by the individuals trained during the “training of trainer” sessions. Due to the heterogeneity of the participants, pre- and post-training assessments were not administered, however post training feedback was obtained using a structured checklist that evaluated how the participants would use the knowledge acquired, potential barriers to use of the acquired knowledge and overall rating of the training. There was an above-average rating of the training offered across the five counties with recommendations to increase duration of the training or tailor sessions to a particular area each time. Figure 3 shows the participants’ rating of the content.

**Figure 4:**
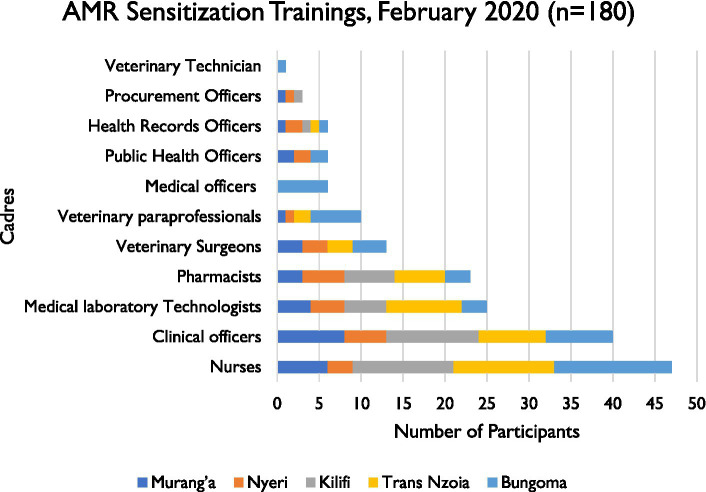
Distribution of healthcare worker’s cadres reached during the counties AMR surveillance sensitizations forums in February 2020.

A total of 180 healthcare professionals were reached. Distribution is shown in Figure 4. In addition to these, other stakeholders implementing AMR-related activities adopted the curriculum and associated materials to build capacity in their respective supported counties. For example, Fleming Fund consortium partners in Kenya trained 15 National Public Health Laboratories staff while a Global Health Security project funded by the U.S. Centers for Disease Control and Prevention conducted on-site trainings in six supported counties.

#### Bacteriology training for laboratory technologists

A hands-on training workshop for medical laboratory technologists was organized that included some of the modules in the AMR surveillance curriculum. Modules giving overview of biosafety and biosecurity measures, pre-analytical processes related to specimen collection, transportation to the laboratory, reception, and storage, laboratory diagnostic processes related to organism identification, antimicrobial susceptibility testing, and results reporting, specimen/isolate referral system, supplies quantification, procurement systems, equipment management processes and quality assurance (QA) in a microbiology laboratory were covered. A total of 15 participants were trained. Participants knowledge and attitudes on AMR and AMR detection were evaluated before and after the training. Pre- and post- training scores were 57.6 and 72.8%, respectively.

## Discussion

The Kenya AMR surveillance curriculum was developed and finalized in a period of less than a year. This is in contrast to the usual curriculum development timelines that can span years (Jippes et al., 2010; Moran et al., 2015; Taneri et al., 2021). At the center of this drive was government commitment to the process and a dedicated team of SMEs from the IDDS project, the Ministry of Agriculture, Livestock, Fisheries and Cooperatives, MOH, research, and academia. Ownership by government meant this was a key priority to them. Engaging a multidisciplinary team of expert stakeholders in government and private sectors, academia brought on board the required expertise to drive the process (Tovar et al., 2012).

Also, in less than 2 years after finalization of the curriculum and the training materials, over 500 healthcare workers have been trained or sensitized using these materials in human sector from 19 of the 47 Kenyan counties. The curriculum materials were quickly adopted by other stakeholders in the country supporting AMR surveillance and used to build capacity in the counties they support. Key among these are partners in Fleming Fund consortium and global health projects funded by the U.S. Centers for Disease Control and Prevention in Kenya. International collaboration has raised awareness of AMR as a global health challenge and spurred actions within the counties to address the threat. Sensitization of clinicians has promoted utilization of bacteriology services, though the volume of requests still falls below targets. While many healthcare workers in the human health sector have been reached, there is need to scale capacity building of healthcare workers in the animal health sector. One-time sensitization forums may not be adequate to drive the desired behavior change in use of antimicrobials among healthcare professionals. Globally, irrational use of antimicrobials has been escalating over several decades (Mboya et al., 2018; Sweileh, 2021); thus, to reverse this trend, investment is needed in regular sensitization and training forums using this standardized curriculum and efforts to achieve a wide coverage (Rogers Van Katwyk et al., 2018; Littlejohn et al., 2019). The ongoing collaborative efforts between MOH and IDDS project to host the curriculum in the MOH virtual academy will increase access to this knowledge through self-paced learning. There is a need for government to develop a comprehensive training evaluation framework for use during scale-up implementation phase, to track course impact in the short and long term and inform improvement efforts to the materials in the future. A cascade approach could be adopted where county-level trainees then train healthcare workers within the counties (Konduri et al., 2017). With the curriculum not designed to replace the pre-service curricula, there is a need for the respective government ministries to continue advocacy with academic institutions to offer competency-based curricula to produce healthcare workers to contribute to national efforts to contain AMR through practices that promote prudent use of antimicrobials.

## Conclusion

The Kenya AMR surveillance curriculum and related training materials are set to standardize capacity building initiatives and facilitate building of competencies required in healthcare professionals for the fight against AMR in Kenya. While the curriculum has been well received, additional investments are needed to reach a tangible representation of healthcare workers in both the human and animal health sectors in the country. A dedicated, multisectoral team of SMEs with government ownership and support of curriculum development experts and professional reviewers and editors can accelerate Kenya’s progress toward AMR prevention and mitigation.

## Data availability statement

The original contributions presented in the study are included in the article/Supplementary material, further inquiries can be directed to the corresponding author.

## Author contributions

JN, JO, SC, DM, and MN primarily created the manuscript with review and input from the AD, AT, ET, EW, RN, RG, SG, and WM. DM handled the project administration. All authors contributed to the article and approved the submitted version.

## Funding

This work was made possible through the support of the United States Agency for International Development (USAID), Global Health under the terms of the Infectious Disease Detection and Surveillance contract GS00Q14OADU119. Views expressed are not necessarily those of USAID or the United States government.

## Conflict of interest

The authors declare that the research was conducted in the absence of any commercial or financial relationships that could be construed as a potential conflict of interest.

## Publisher’s note

All claims expressed in this article are solely those of the authors and do not necessarily represent those of their affiliated organizations, or those of the publisher, the editors and the reviewers. Any product that may be evaluated in this article, or claim that may be made by its manufacturer, is not guaranteed or endorsed by the publisher.
