# Supplementary material for: Development, roll-out and implementation of an antimicrobial resistance training curriculum harmonizes delivery of in-service training to healthcare workers in Kenya
Source: Front Microbiol. 2023 Aug 1;14:1142622. doi: 10.3389/fmicb.2023.1142622 (PMC10427499; doi:10.3389/fmicb.2023.1142622)
Supplement: Supplementary file 1 [file Presentation_1.pdf]

REPUBLIC OF KENYA

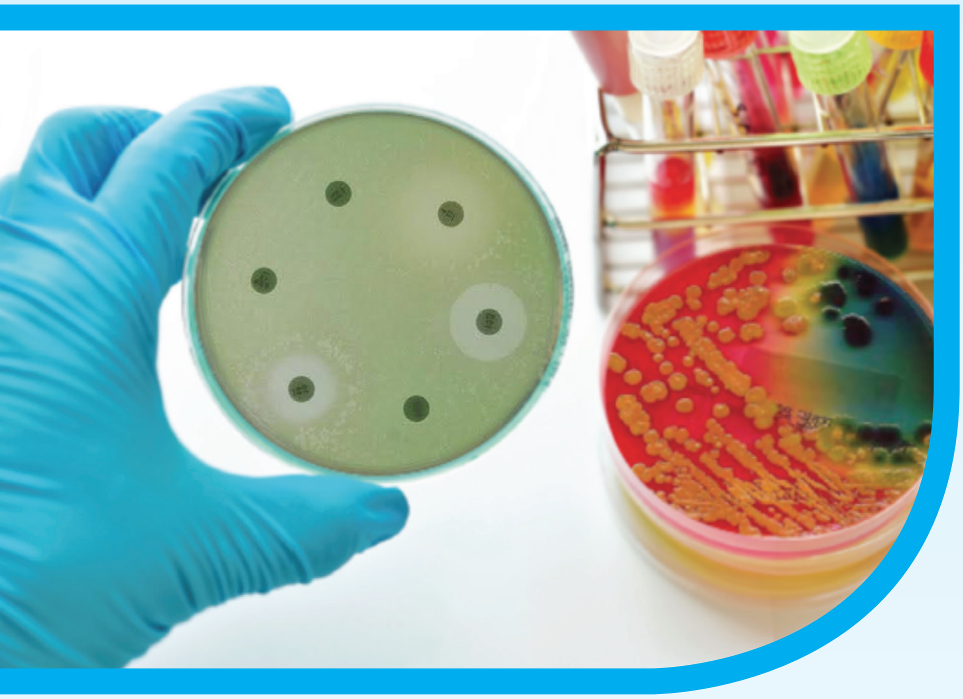

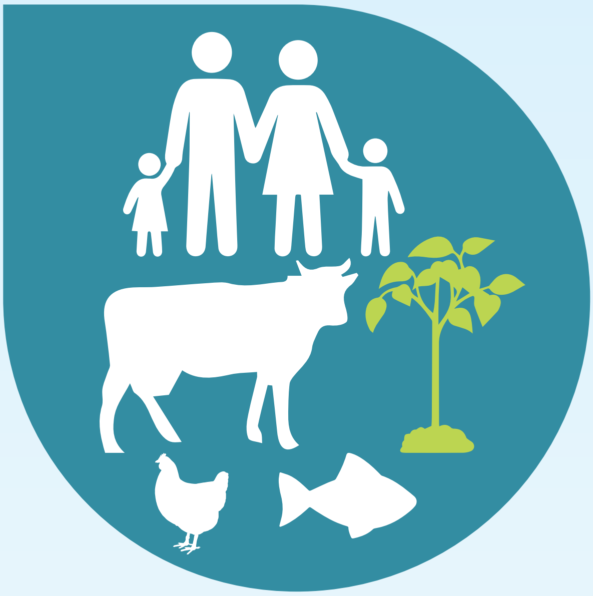

NATIONAL ANTIMICROBIAL RESISTANCE SURVEILLANCE TRAINING CURRICULUM

JANUARY 2020

This is a publication of the Ministry of Health/Ministry of Agriculture Livestock and Fisheries.

Any part of this document may be freely reviewed, quoted, reproduced, and translated in full or in part, provided the source is acknowledged. It may not be sold or used for commercial purpose or profit.

Antimicrobial Resistance Surveillance Training Curriculum 2020.

TABLE OF CONTENTS

Abbreviations and acronyms iii

Forward IV

Acknowledgement v

INTRODUCTION 1

Rationale for the AMR Surveillance Training Curriculum 2

Organization of the Curriculum 2

Target Audience 2

Summary of the Modules 3

Course objectives 4

Course Duration 4

MODULE 1: OVERVIEW OF ANTIMICROBIAL RESISTANCE, DIAGNOSIS, AND SURVEILLANCE 5

1.1 Overview of AMR 5

1.2 Kenya National Policy and Action Plan on the prevention and containment of Antimicrobial Resistance 5

1.3 Summary of the National AMR Surveillance Strategy 6

1.4 Introduction to Diagnostic Stewardship 6

1.5. Introduction to Antimicrobial Stewardship 7

1.6 Introduction to Infection Prevention and Control 7

MODULE 2: DRIVERS OF ANTIMICROBIAL RESISTANCE AT THE HUMAN-ANIMAL-ENVIRONMENT INTERFACE 8

MODULE 3: INTRODUCTION TO BIOSAFETY AND BIOSECURITY 9

MODULE 4: SPECIMEN COLLECTION, TRANSPORT, RECEPTION, AND STORAGE 10

4.1 Specimen Collection 10

4.2 Packaging and Transport 10

4.3 Specimen Receipt 11

4.4 Storage 11

4.5 Custody of Samples and Isolates 11

MODULE 5: MICROBIOLOGICAL PROCEDURES.......... 12
5.1 Basic Microscopy.......... 12
5.2 Routine Microscopy Procedures: Wet Mount, Smear Preparation, Gram Stain Principle, Method, Interpretation, and Quality Control.......... 13
5.3 Culture Media: Preparation, Storage, and Quality Control.......... 13
5.4 Specimen Processing.......... 13
5.5 Culture Reading and Bacterial Identification Tests.......... 14
5.5.1 Gram-Positive Organism Identification.......... 15
5.5.2 Gram-Negative Organism Identification.......... 15
5.6 Antibiotic Susceptibility Testing.......... 16
5.7 Molecular Testing for Bacterial Identification and AMR.......... 17
MODULE 6: SPECIMEN REFERRAL AND REPORTING.......... 18
MODULE 7: QUALITY ASSURANCE IN THE CLINICAL MICROBIOLOGY LABORATORY.......... 19
MODULE 8: AMR SURVEILLANCE MONITORING, EVALUATION, AND REPORTING.......... 20
8.1 AMR Surveillance.......... 20
8.2 Data and Information Management.......... 20
8.3 AMR Data Analysis and Reporting.......... 21
8.4 Integrated AMR Surveillance, One Health Approach.......... 21
8.5 Utilization of AMR Surveillance Data.......... 22
MODULE 9: PROCUREMENT AND SUPPLY CHAIN MANAGEMENT.......... 23
MODULE 10: EQUIPMENT MANAGEMENT.......... 24
MODULE 11: CLINICAL GUIDE.......... 25
MODULE 12: PRUDENT USE OF ANTIMICROBIALS IN VETERINARY PRACTICE.......... 26
12.1 Use of Antimicrobials.......... 26
12.2 Policy and Legislation in Antimicrobial Use.......... 27
COURSE EVALUATION AND DELIVERY METHODS.......... 28
RESOURCES/LOGISTICS REQUIREMENTS.......... 28
REFERENCES.......... 29
COURSE ORGANIZATION.......... 30
LIST OF CONTRIBUTORS.......... 32

Abbreviations and acronyms

**AMP**	Assessment, Mitigation, and Performance
**AMR**	Antimicrobial Resistance
**AMS**	Antimicrobial Stewardship
**AMU**	Antimicrobial Use
**AST**	Antimicrobial Susceptibility Testing
**BSC**	Biosafety Cabinet
**CAPA**	Corrective Action Preventive Action
**CEC**	County Executive Committee Members
**CLSI**	Clinical and Laboratory Standards Institute Guidelines
**CMLC**	County Medical Laboratory Coordinator
**EQA**	External Quality Assurance
**GHSA**	Global Health Security Agenda
**ID**	Organism Identification
**IHR**	International Health Regulations
**IQC**	Internal Quality Control
**JEE**	Joint External Evaluation
**LIS**	Laboratory Information System
**MALF**	Ministry of Agriculture, Livestock and Fisheries
**MIC**	Minimum Inhibitory Concentration
**MoH**	Ministry of Health
**MSDS**	Material Safety Data Sheet
**NASIC**	National Antimicrobial Stewardship Interagency Committee
**NPHLS**	National Public Health Laboratories
**NMRL**	National Microbiology Reference Laboratory
**OIE**	World Organization for Animal Health
**PEP**	Post Exposure Prophylaxis
**PPE**	Personal Protective Equipment
**PT**	Proficiency Testing
**QA**	Quality Assurance
**QC**	Quality Control
**SOP**	Standard Operating Procedure
**CSF**	Cerebrospinal Fluid
**DNA**	Deoxyribonucleic Acid
**NGS**	Next Generation Sequencing
**PCR**	Polymerase Chain Reaction
**LAMP**	Loop-Mediated Amplification
**WGS**	Whole Genome Sequencing
**CQI**	Continuous Quality Improvement
**CAC**	Codex Alimentarius Commission
**RCP**	Recommended Code of Practice

Forward

Antimicrobial Resistance (AMR) is becoming a global health concern in the human and animal health sectors and the environment. It occurs when a microorganism develops resistance to an antimicrobial drug to which it was previously sensitive. The increasing resistance is due primarily to misuse of antimicrobials in both human and animals.

The World Health Organization (WHO), the World Organization for Animal Health (OIE), and the United Nations Food and Agricultural Organization have taken measures to guide their Member States on measures to prevent and control antimicrobial resistance. In 2015, WHO developed a Global Action Plan (GAP) to prevent and control AMR and this required member states to develop a National Action Plan (NAP) aligned to the Global Action Plan.

Following the development of GAP, the Government of Kenya has developed National Policy and Action Plan on AMR and has established a multi-sectoral National Antimicrobial Stewardship Interagency Committee (NASIC) to coordinate actions against AMR.

AMR surveillance is one of the objectives of the Kenya National Action Plan. Other objectives include the need to increase knowledge and awareness of AMR, Antimicrobial Stewardship, Infection Prevention and Control, and research and development. In line with the national policy and action plan, and to inform policy decisions and improve clinical outcomes, the human and animal health sectors have developed surveillance strategies to guide actions for detecting, reporting, and monitoring trends in Kenya.

The development of this document provides a timely tool to guide systematic strengthening of knowledge and skills development among all individuals involved in AMR prevention and control including leadership and governance, clinical practice and laboratory detection, and surveillance data management in both the human and animal health sectors.

Dr. Patrick Amoth
Ag. Director General for Health
Ministry of Health.

Dr. Obadiah N. Njagi , PHD, OGW
Director of Veterinary Services
Ministry for Agriculture, Livestock and Fisheries

Acknowledgement

The successful development of this document was possible due to close partnership and collaboration among multiple national and international organizations and individuals across different sectors and disciplines, and under the leadership of the Government of Kenya Ministry of Health's National Antimicrobial Resistance Stewardship Interagency Committee Secretariat. We sincerely appreciate the selfless commitment of these individuals and organizations in devoting their time and resources to this noble undertaking.

The active collaboration across human and animal health sectors and disciplines ensured that this document was developed using a One Health approach and the contents recognize and integrate the contribution and value of all sectors of government. We wish to thank our colleagues from the Ministry of Agriculture, Livestock and Fisheries and allied institutions for their contributions.

We particularly wish to thank the USAID-funded Infectious Disease Detection and Surveillance Project for the logistical and technical assistance accorded to the Government throughout this process. We also thank other multilateral and bilateral development partners for the pivotal roles they played through their resources and expertise.

Finally, we wish to recognize the coordination and leadership roles played by government officials from the National AMR Secretariat in the Directorate of Standards, Quality Assurance, Policy and Regulation, and National Public Health Laboratory Services in the Ministry of Health (MoH). Also, the Ministry of Agriculture, Livestock & Fisheries for their valuable contribution and being part of the process. We specifically acknowledge the critical roles played by Dr. Evelyn Wesangula, the National AMR Secretariat focal person in the MoH, and Susan Githii, the National AMR Surveillance focal person in the National Public Health Laboratory, for their leadership.

Dr. Simon K. Kibias, OGW
Head, Directorate of Standards, Quality Assurance, Policy & Regulation
Ministry of Health.

Dr. Charles Ochodo, PhD
Senior Deputy Director of Veterinary Services
Ministry of Agriculture, Livestock and Fisheries.

INTRODUCTION

Antimicrobial Resistance (AMR) is a growing global concern for the public health, animal health and the environment. Globally, it is projected that by 2050, the health consequences and economic costs of AMR will result in 10 million human fatalities annually and a 2–3.5 percent decrease in global gross domestic product. Several studies and reports in Kenya have documented the increasing trend of antimicrobial use and resistance in both humans and animals. The actual trend and exact burden is, however, unknown due to lack of systematic surveillance[1].

The International Health Regulations (IHR) Joint External Evaluation (JEE) conducted in 2017 showed that although Kenya has made some progress in strengthening the country's capacity to prevent, detect, report, and respond to public health threats, significant gaps remain[2]. The implementation status of each core capacity is indicated by a score, which reflects the Kenya's level of advancement, its capacity to institutionalize technical area competencies, and ensure that they are sustainable. Score 2 indicates a Limited capacity with attributes of a capacity being in development stages (implementation has started with some attributes achieved and others commenced). Score 3 indicates a developed capacity in place, however a sustainability plan has not been ensured (such as through inclusion in the operational plan of the National Health Sector Plan with a secure funding source). Score 4 demonstrates capacity and attributes are in place and sustainable for a few years and can be measured by the inclusion of attributes or IHR core capacities in the National Health Sector Plan and a secure funding source.

Table 1 below provides a snapshot of the capacity scores as at 2017 baseline in select indicators across AMR, the National Laboratory System, and surveillance technical areas.

Table 1: 2017 JEE select indicators scores for Kenya[2].

JEE technical area/action package	Indicator	Score
Antimicrobial resistance (AMR)	Prevent: 3.2 Surveillance of AMR	2
National Laboratory System	Detect: 1.2 Specimen referral and transport system	2
Surveillance	Detect: .2.2 Use of electronic tools	2
Surveillance	Detect: .2.3 Analysis of surveillance data	4

In recognition of the growing threat of AMR, in 2015 the World Health Organization (WHO) developed a Global Action Plan on AMR following a 2014 resolution on AMR at the World Health Assembly (WHA 67.25) which called on the agency to develop a draft global action plan to ensure that all countries have the capacity to combat AMR[3]. In 2017, the Kenya Ministries responsible for Health and Agriculture spearheaded the development of a National Policy and Action Plan to combat AMR, in line with the WHO's Global Action Plan and IHR[4]. Subsequently the sectors set up a multi-sectoral National Antimicrobial Stewardship Interagency Committee (NASIC) to coordinate efforts across sectors and partners.

In 2018, the Ministry of Health (MoH) and the Ministry of Agriculture and Livestock and Fisheries (MALF) worked with NASIC to develop AMR surveillance strategies to guide systematic AMR detection and data collection and management in their respective sectors[5]. Since 2018, NASIC has piloted the AMR surveillance strategy in the human health sector in two counties, with the aim of increasing the number of surveillance sites to 28 by 2022. Knowledge and skills development of laboratory diagnostics and data and information management is at the core of surveillance system strategy and NASIC plans to standardize AMR surveillance training through the development of this training curriculum.

Rationale for the AMR Surveillance Training Curriculum

The National Policy on prevention and containment of AMR outlines the need to strengthen the knowledge and evidence base for AMR, optimizing the use of antimicrobials in human, animal, and plant health and support for new medicines, diagnostic tools, and other interventions[1].

In-service health workers' knowledge and skills development is critical for the generation and management of AMR surveillance data which are the foundation of quality diagnostics. Therefore, training and re-training of health care workers is a core part of the capacity-building package for all new surveillance sites. Currently, no standard training course exists to guide AMR surveillance in-service training in Kenya, and NASIC has prioritized the development of a standard training course on AMR surveillance to guide training of National and County teams. This curriculum is expected to act as a guide for all stakeholders conducting AMR surveillance in the country to ensure uniform comprehensive and systematic capacity building.

Organization of the Curriculum

The curriculum is organized into modules, each focusing on a specific area based on (a) the need to share information on AMR, or (b) the need to transfer knowledge and skills on AMR diagnosis and surveillance.

Module 1 gives a global and national overview of AMR and a summary of the measures and strategies Kenya is using to combat AMR. Module 2 introduces participants to AMR occurrence at the human-animal -environment interface and the need for a One Health approach. Module 3 provides an overview of biosafety and biosecurity measures related to AMR diagnostics. Module 4 addresses pre-analytical processes related to specimen collection, transportation to the laboratory, reception, and storage.

Module 5 focuses on the laboratory diagnostic processes related to Organism Identification, Antimicrobial Susceptibility Testing, and results reporting. Module 6 focuses on the sample/isolate referral system and Module 7 deals with Quality Assurance in a microbiology laboratory. Module 8 focuses on all aspects of AMR surveillance monitoring, evaluation, reporting, and data management. Modules 9 and 10 address all aspects of supplies forecasting, procurement systems, and equipment management processes. Modules 11 and 12 deal with the need for a guide for clinicians in microbiology testing and the prudent use of antimicrobials in the human and animal health sectors.

Target Audience

The curriculum is targeted to all stakeholders and health care workers who play various roles in AMR diagnostics and surveillance, including clinicians, veterinarians, veterinary paraprofessionals, laboratory personnel, pharmacists, nurses, and records officers. Those responsible for AMR governance and leadership and resource mobilization and allocation, especially at the county levels, are also included.

Different categories within the target audience will undertake specific modules based on their needs, as indicated below:

- Laboratory practitioners —All modules.
- Clinicians—Pre-analytical, Analytical and Post-analytical modules.

- Veterinarians—Pre-analytical, Analytical, and Post-analytical modules.
- Nurses—Pre-analytical and Post-analytical modules.
- Pharmacists—Analytical and Post-analytical modules.
- Records (IT, bioinformatics, clerks)—Pre-analytical, Analytical and Post-analytical modules.
- Biomedical Engineers—Analytical modules.
- County Leadership (CECs health and agriculture, County Directors of Health and Veterinary Services, CMLCs, Med Sups)—Modules 1, 2, 11, and 12.

Summary of the Modules

Pre analytic

- Module 1: Overview of Antimicrobial Resistance Diagnosis and Surveillance.
- Module 2: Drivers of Antimicrobial Resistance at the Animal-Environment-Human Interface.
- Module 3: Introduction to Biosafety and Biosecurity.
- Module 4: Specimen Collection, Transport, Reception, and Storage.
- Module 6: Specimen Referral and Reporting.
- Module 7: Quality Assurance in the Clinical Microbiology Laboratory.
- Module 9: Procurement and Supply Chain Management.
- Module 10: Equipment Management.
- Module 11: Clinical Guide.
- Module 12: Prudent use of Antimicrobials in Veterinary Practice.

Analytic

- Module 5: Microbiological Procedures.
- Module 7: Quality Assurance in the Clinical Microbiology Laboratory.

Post analytic

- Module 4: Specimen Collection, Transport, Reception, and Storage.
- Module 8: AMR Surveillance Monitoring, Evaluation, and Reporting.
- Module 6: Specimen Referral and Reporting.
- Module 7: Quality Assurance in the Clinical Microbiology Laboratory.
- Module 11: Clinical Guide.
- Module 12: Prudent Use of Antimicrobials in Veterinary Practice.

Course objectives

The course is designed to have objectives at two levels. One is participants taking pre-analytic module alone. The other one is for the participants taking analytic and post analytic modules.

The pre analytic modules of the course are designed with an objective of equipping participants with the knowledge and skills to:

- Develop and deploy customized AMU/AMR containment and prevention interventions.
- Apply a One Health approach in addressing AMR agenda.
- Coordinate, monitor and evaluate multi-sectoral stewardship activities targeted at mitigating against AMR.

The analytic and post analytic modules of the course is designed with an objective of equipping learners with the knowledge and skills to:

- Utilize advances in diagnostic techniques (e.g., rapid molecular tests, biomarkers) and design methods to incorporate their use/application to stewardship initiatives.
- Design and deploy AMR Surveillance Monitoring, Evaluation, and Reporting.
- Use antimicrobials judiciously to minimize or eliminate occurrence of AMR.

Course Duration

The recommended course duration is five days for National Training of Trainers (TOT), two days for county-based health care workers and five days for laboratory practitioners. The modular approach ensures that it can be tailored to address various audiences and needs.

MODULE 1: OVERVIEW OF ANTIMICROBIAL RESISTANCE, DIAGNOSIS, AND SURVEILLANCE

Purpose:

The purpose of this module is to provide an overview of antimicrobial resistance (AMR), diagnosis, surveillance, stewardship programs, and the global and national policy frameworks for combating AMR.

Target Audience: Clinicians, veterinarians, nurses, veterinary paraprofessionals, pharmacists, pharmacy practitioners, laboratory practitioners, public health officers, records officers, biomedical engineers, county leadership (Chief Officers of Health and of Agriculture, Chair of Health & Agriculture Committees, County Executive Committee Member for Health and Agriculture, County Directors of Health and Veterinary Services, County Medical Laboratory Coordinators, Medical Superintendent) and any other relevant stakeholder or officer.

Expected learning outcome for the module:

By the end of this module, participants will:

- Design and implement in-service education on mitigation and prevention of AMR for stakeholders in healthcare.
- Identify AMR and formulate intervention programs in line with One Health approaches.

1.1 Overview of AMR

Expected Learning Outcomes:

By the end of this module, participants will be able to:

- Define terms commonly used in antimicrobial resistance.
- Classify antimicrobial agents and describe mode of action.
- Define AMR and its causes.
- Outline the global, regional, and national perspective and impact of AMR.
- Describe the International Health Regulations (IHR 2005) and AMR prevention and containment.

Content Outline:

- Definition of terms used in AMR.
- Antimicrobial classification and mode of action.
- Antimicrobial resistance and its causes.
- Global, regional, and national perspective and impact of AMR.
- International Health Regulations 2005 and the AMR prevention and containment.

1.2 Kenya National Policy and Action Plan on the prevention and containment of Antimicrobial Resistance

Purpose: To provide an overview of Kenya National Policy Frameworks to combat AMR

Expected Learning Outcomes:

By the end of this module, participants will be able to:

- Describe the scope and objectives of the National Policy and Action Plan on prevention and containment of AMR in Kenya.
- Explain the national and county leadership and governance structures for combating AMR as outlined in the National Action Plan.
- Define the roles and responsibilities of stakeholders in AMR.
- Outline the AMR National Action Plan monitoring and evaluation plan.

Content Outline:

- Overview of the national policy and action plan to combat AMR in Kenya.
- Objectives and scope of the National Policy and Action Plan on prevention and containment of AMR in Kenya.
- AMR leadership and governance structures as described in the AMR National Action Plan
- Roles and responsibilities of AMR stakeholders.
- AMR National Action Plan monitoring and evaluation plans.

1.3 Summary of the National AMR Surveillance Strategy

Purpose: To provide an overview of the National Antimicrobial Resistance (AMR) surveillance strategy.

Expected Learning Outcomes:

By the end of this module, participants will be able to:

- Outline the goal and objectives of the National AMR surveillance strategy.
- Define the roles and responsibilities of stakeholders in AMR surveillance.
- Outline AMR surveillance quality and reporting standards.

Content Outline:

- Goals and objectives of national AMR surveillance strategy.
- Roles and responsibilities of stakeholders in AMR surveillance.
- AMR surveillance quality and reporting standards.

1.4 Introduction to Diagnostic Stewardship

Purpose: To provide an overview of Diagnostic Stewardship to contain and prevent AMR.

Intended Learning Outcomes:

By the end of this module, participants will be able to:

- Define Diagnostic Stewardship.
- Illustrate the steps and elements of Diagnostic Stewardship.
- Describe the organizational aspects of Diagnostic Stewardship.
- Discuss the barriers to Diagnostic Stewardship.

Content Outline:

- Diagnostic Stewardship.
- Steps and elements of Diagnostic Stewardship.

- Organizational aspects of Diagnostic Stewardship.
- Barriers to Diagnostic Stewardship.

1.5 Introduction to Antimicrobial Stewardship

Purpose: To provide an overview of the National Guidelines on Antimicrobial Stewardship (AMS).

Expected Learning Outcomes:

By the end of this module, participants will be able to:

- Outline the National Guidelines on Antimicrobial Stewardship (AMS).
- Define Antimicrobial Stewardship.
- Outline the goals of an AMS program.
- State the steps and core elements of an AMS program.
- Describe how to establish an AMS program.
- Summarize potential pitfalls and mitigation strategies.

Content Outline:

- Overview of National Guidelines on Antimicrobial Stewardship (AMS).
- Antimicrobial Stewardship.
- Goals of an AMS program.
- Steps and core elements of an AMS program.
- How to establish an AMS program.
- Potential pitfalls and mitigation strategies for an AMS program.

1.6 Introduction to Infection Prevention and Control

Purpose: To provide an overview of Infection Prevention and Control (IPC) in a One Health context and the inter-relationship with AMR prevention and control.

Expected Learning Outcomes:

By the end of this module, participants will be able to:

- Define "Infection Prevention and Control (IPC)" and "Healthcare-Associated Infections (HAIs)".
- Describe the historic evolution of IPC.
- Describe the chain of infection.
- Explain the benefits of adherence to IPC practices.
- Describe structures for management and coordination of IPC.

Content Outline:

- "Infection Prevention and Control (IPC)" and "Healthcare-Associated Infections (HAIs)".
- Historic evolution of IPC.
- The chain of infection.
- Benefits of adherence to IPC practices.
- Structures for management and coordination of IPC.

MODULE 2: DRIVERS OF ANTIMICROBIAL RESISTANCE AT THE HUMAN-ANIMAL-ENVIRONMENT INTERFACE

Purpose:

The purpose of this module is to provide participants with knowledge of drivers of antimicrobial resistance at the human-animal-environment interface.

Target Audience : Laboratory practitioners, clinicians, veterinarians, nurses, pharmacy practitioners, public health officers, record personnel and any other relevant stakeholder or officer.

Expected Learning Outcomes:

By the end of this module, participants will be able to:

- Describe the contribution of humans toward the emergence of antimicrobial resistance.
- Explain the relevance of animal production and environmental factors in the emergence of antimicrobial resistance.
- Describe the mechanism of the spread of antimicrobial resistance between animals, humans, and the environment.
- Explain the significance of the One Health approach in the fight against antimicrobial resistance.

Content Outline:

- Contribution of humans toward the emergence of antimicrobial resistance.
- Relevance of animal production in the emergence of antimicrobial resistance.
- Mechanism of the spread of antimicrobial resistance between animals, the environment, and humans.
- Significance of the One Health approach in the fight against antimicrobial resistance.

MODULE 3: INTRODUCTION TO BIOSAFETY AND BIOSECURITY

Purpose:

The purpose of this module is to provide participants with knowledge, skills, and an understanding of the urgency of ensuring that biosafety and biosecurity procedures are established and observed for a safe and secure work environment.

Target Audience:All healthcare workers (Laboratory practitioners, clinicians, veterinarians, nurses, pharmacists, public health officers, records personnel) and any other relevant stakeholder or officer.

Expected Learning Outcomes:

By the end of this module, participants will be able to:

- Define key terms in bio-risk management.
- Describe different types of hazards.
- Outline elements of bio-risk management- Assessment Mitigation Performance (AMP) model.
- Describe steps in bio-risk assessment: identification of the hazard, characterization and evaluation of bio-risk.
- Identify the five categories of mitigation measures for controlling biological risks.
- Describe Bio-risk Performance.

Content Outline:

- Definition of terms: laboratory biosafety, laboratory biosecurity, field biosafety and biosecurity, Valuable Biological Materials (VBM), Valuable Laboratory Materials (VLM), bio-risk and bioethics, biohazards, post exposure prophylaxis (PEP), and personal protective equipment (PPE).
- Types and sources of hazards: physical, mechanical, electrical, radiation, chemical, and biological; biosafety levels and risk group classification.
- Elements of bio-risk management: bio-risk assessment, bio-risk mitigation, and bio-risk performance (AMP model).
- Bio-risk assessment: biohazard identification, bio-risk characterization and evaluation.
- Bio-risk mitigation.
    - ◊ Hierarchy of Controls (elimination, substitution, engineering, administrative, and PPE).
    - ◊ Laboratory Biosecurity Pillars: physical biosecurity, materials and accountability, personnel security, information security, and transport security.
    - ◊ Good Laboratory Work Practices (standard precautions, PEP, PPE, spills and spill kits).
    - ◊ Disinfection and Disinfectants: definition, methods of disinfection, including boiling, chemical disinfectants, mode of action, choice of disinfectants, disinfection of work areas.
    - ◊ Sterilization: definition, methods of sterilization, and indicators of sterilization
    - ◊ Microbiological Waste Management: segregation, collection, storage, transportation, treatment and disposal.

- Performance: role of performance in bio-risk management.
- Biosafety and Biosecurity: Case studies and exercises.

MODULE 4: SPECIMEN COLLECTION, TRANSPORT, RECEPTION, AND STORAGE

Purpose:

The purpose of this module is to provide participants with the knowledge and skills for proper collection, packaging, transport, receipt of specimens, sample acceptance and rejection criteria, storage, and chain of custody for microbiology testing.

Target Audience: Laboratory practitioners, clinicians, other persons involved in collection and transportation of samples and any other relevant stakeholder or officer.

4.1 Specimen Collection

Expected Learning Outcomes:

By the end of this module, participants will be able to:

- Describe the specimen types needed to diagnose priority bacterial pathogens.
- Illustrate how to aseptically collect specimen to detect priority AMR pathogens.
- Explain how to select suitable containers for the collection of different specimens.
- Properly label specimens to be submitted to the microbiology laboratory.
- Explain the importance of timely communication between the microbiology laboratory and those collecting specimens.
- Discuss rules and principles that must be followed in order to collect microbiology specimens that accurately reflect the causative microbiological agent.
- Describe the rejection criteria for microbiological samples.

Content Outline:

- Rules and principles of appropriate microbiology specimens.
- Specimen types needed to diagnose priority bacterial pathogens.
- Suitable containers for the collection of different specimens.
- Proper collection of the specimens.
- Proper labelling of specimens to be submitted to the microbiology laboratory.

4.2 Packaging and Transport

Expected Learning Outcomes:

By the end of this module, participants will be able to:

- Describe the general instruction on packaging and triple packaging materials.
- Explain the proper transportation of specimens including the different transport media used.
- Describe temperature and time requirements for the transportation of microbiological specimens.
- Define how to transport specimens for laboratory testing within and out of the collection center.

Content Outline:

- General instructions on packaging and triple packaging materials.
- Transport media used in microbiology.

- Temperature and time requirements for transportation of microbiology specimens.
- Transportation of specimens for lab testing within and out of the collection center.
- Transportation of specimens and isolates: Shippers Declaration Form.

4.3 Specimen Receipt

Expected Learning Outcomes:

By the end of this module, participants will be able to:

- Describe the information that must be on each specimen and requisition form in order to be accepted into the laboratory for testing.
- Describe the procedure for specimen reception and filing in the laboratory register/laboratory information system (LIS).
- Describe specimen acceptance and rejection criteria for all microbiology specimens based on established guidelines.

Content Outline:

- Information on the specimen and laboratory test requisition form.
- Procedure for specimen reception and filing in the laboratory register/LIS.
- Microbiology specimen rejection criteria.

4.4 Storage

Expected Learning Outcomes:

By the end of this module, participants will be able to:

- Describe procedures for microbiology specimen storage before and after processing.
- Describe common pitfalls in specimen storage.
- Explain proper storage of high priority isolates for long-term viability.

Content Outline:

- Priority bacterial infectious diseases specimen storage requirements before and after processing.
- Common pitfalls in specimen storage.
- Proper storage of high priority isolates for long-term viability.

4.5 Custody of Samples and Isolates

Expected Learning Outcomes:

By the end of this module, participants will be able to:

- Describe how the laboratory implements chain of custody.
- Describe tools used for chain of custody.

Content Outline:

- Steps to implement chain of custody.
- Tools used for chain of custody.

MODULE 5: MICROBIOLOGICAL PROCEDURES

5.1 Basic Microscopy

Purpose: To ensure personnel have basic skills in the use, care, and maintenance of a microscope

Target Audience: Laboratory practitioners, veterinarians, veterinary paraprofessionals any other relevant stakeholder or officer.

Expected Learning Outcomes:

By the end of this module, participants will be able to:

- Identify different parts of a light microscope.
- Demonstrate skills on the use of a microscope.
- Perform routine maintenance of a microscope.

Content Outline:

- Parts of a light microscope.
- Proper use of a microscope.
- Routine maintenance of a microscope.

5.2 Routine Microscopy Procedures: Wet Mount, Smear Preparation, Gram Stain Principle, Method, Interpretation, and Quality Control

Purpose: To equip laboratory practitioners with the skills required for smear preparation and interpretation.

Expected Learning Outcomes:

By the end of this module, participants will be able to:

- Prepare a wet mount.
- Interpret the appearance of cells (normal and infected cells) and commonly found organisms.
- Describe the purpose and principle of a Gram stain.
- Identify the types of reagents used in a Gram stain procedure.
- Prepare smears for Gram staining from clinical specimens and cultures.
- Describe the steps followed in Gram stain procedure.
- Describe the differences between Gram-positive and Gram-negative bacteria.
- Describe the morphological appearance based on Gram stain reaction.
- Prepare a set of slides to be used for Gram stain quality control (QC).

Content Outline:

- Preparation and interpretation of a wet mount (India ink, Nigrosin, Lactophenol blue)
- Gram Staining:
    - ◊ Purpose and principle of the Gram stain.
    - ◊ Types of reagents used in the Gram stain procedure.
    - ◊ Preparation of smears for Gram staining from clinical specimens and cultures.
    - ◊ Steps in the Gram stain procedure.

- ◊ Differences between Gram-positive & Gram-negative bacteria.
- ◊ Morphological appearance based on Gram stain reaction; appearance of human and/or animal cells associated with infectious processes.
- ◊ Preparation of Gram stain QC slides.

5.3 Culture Media: Preparation, Storage, and Quality Control

Purpose: To equip laboratory practitioners with skills for culture media preparation, storage, and quality control.

Expected Learning Outcomes:

By the end of this module, participants will be able to:

- Differentiate types of culture media.
- Describe the proper procedure for media preparation, dispensing, and documentation.
- Label media appropriately.
- Describe characteristics of good quality culture media.
- Identify commonly encountered problems with basic culture media.
- Select appropriate storage requirements for each media type.
- Apply expiration dating guidelines for each media type.
- Carry out quality control of culture media.

Content Outline:

- Types of media.
- Procedure for media preparation and proper documentation.
- Appropriate media labeling.
- Suitable characteristics of good quality culture media.
- Commonly encountered problems with basic culture media.
- Storage requirements for each media type.
- Culture media quality control:
    - ◊ Perform media QC and proper documentation.
    - ◊ Selection of appropriate QC organisms.
    - ◊ Inoculation of media for QC testing.
    - ◊ Maintenance of and subculture stock cultures used for QC.

5.4 Specimen Processing

Purpose: To equip personnel with skills on specimen processing.

Expected Learning Outcomes:

By the end of this module, participants will be able to:

- Describe procedure for ruling out anthrax in all specimens (carcasses) submitted to the laboratory. (Applicable to veterinary laboratory personnel only for specimens of ruminant origin).
- Describe the methods used for the primary culture for common types of specimens received in the microbiology laboratory, including choice of correct media, incubation temperatures, atmospheres, and duration.

- Explain the requirements for direct microscopy for each specimen type and describe and interpret the smears appropriately.
- Describe the culture media needed for different specimen types and the inoculation technique.

Content Outline:

- Procedure for ruling out anthrax in all carcasses submitted to the laboratory (Describe ingredients of good quality culture media).
- Methods used for the primary culture for specimens received in the microbiology laboratory, including choice of correct media, incubation temperatures, atmospheres, and duration.
- Requirements for direct microscopy for each specimen type and how to describe and interpret the smears appropriately.
- Inoculation technique required for different specimen types.

5.5 Culture Reading and Bacterial Identification Tests

Purpose: To equip participants with knowledge and skills to perform identification and interpretation of culture results from different specimen types, including blood, cerebrospinal fluid (CSF), sputum, urine, stool, aspirates, tissues and other exudates.

Expected Learning Outcomes:

By the end of this session, participants will be able to:

- List common pathogens isolated from different body sites.
- List organisms found as normal flora from different body sites.
- Interpret cultures that contain normal flora, determine the potential pathogens per specimen type, and accurately report results.
- Quantify growth on primary plating media.
- Describe gross colonial morphology of bacterial growth on primary plating media.
- Identify potential pathogens and normal flora per specimen type.
- Recognize plate contamination.
- Prepare smears from culture colonies for Gram stain.
- Use the appropriate flowchart to make decisions about organism identification.
- Determine identification tests appropriate for the suspected pathogen.
- Prepare a pure culture of the potential pathogen for further testing and stocking.
- Correlate direct Gram stain and culture results.
- Document all observations and results on culture worksheets.
- Perform and interpret common bacterial identification tests (manual biochemical tests, Analytical Profile Index ID, and automated systems).
- Identify organisms (genus and species) based on test results, Gram stain, and colonial morphology and perform rapid serotyping.
- Perform and interpret QC results for all identification test reagents.
- Issue appropriate preliminary and final reports as per standing operating procedure (SOP).

Content Outline:

- Common pathogens isolated from different body sites.
- Organisms found as normal flora from different body sites.
- Interpretation of cultures that contain normal flora, workup of potential pathogens per specimen type, and reporting.
- Quantification of growth on primary plating media.
- Gross colonial morphology of bacterial growth on primary plating media.
- Recognizing plate contamination.
- Colony preparation for Gram stain from cultures.
- Flowchart to make decisions about organism identification.
- Identification tests appropriate for the suspected pathogen.
- Pure culture preparation of potential pathogen for further testing.
- Perform and interpret QC results for all identified test reagents.
- Appropriate preliminary and final reports as per SOP.

5.5.1 Gram-Positive Organism Identification

Purpose: To equip laboratory practitioners with knowledge on how to read a Gram stain, how to identify colonial characteristics, and biochemical tests and other testing algorithms used to identify Gram-positive microorganisms.

Expected Learning Outcomes:

By the end of this module, participants will be able to:

- Identify different types of bacterial morphology and colonial characteristics.
- Use flowcharts and identification charts to identify common aerobic Gram-positive microorganisms.
- Associate various biochemical tests with their correct applications and interpret biochemical test results.

Content Outline:

- Bacterial morphology seen on a Gram stain reaction.
- Different types of colonial characteristics.
- Flowcharts and identification charts for common Gram-positive microorganisms.
- Results of biochemical methods and interpretation.

5.5.2 Gram-Negative Organism Identification

Purpose: To familiarize laboratory professionals on how to read a Gram stain, identify colonial characteristics, biochemical tests used to identify Gram-negative microorganisms, and commonly used testing algorithms.

Expected Learning Outcomes:

By the end of this module, participants will be able to:

- Identify different types of bacterial morphology and colonial characteristics.
- Use flowcharts and identification charts to identify some common aerobic Gram-negative microorganisms.
- Associate various biochemical tests with their correct applications and interpret biochemical test results.

Content Outline:

- Bacterial morphology seen on a Gram stain reaction.
- Different types of colonial characteristics.
- Flowcharts and identification charts for common Gram-negative microorganisms.
- Results of biochemical methods and interpretation.

5.6 Antibiotic Susceptibility Testing

Purpose: To equip personnel with skills in the performance, interpretation, and quality control (QC) of antimicrobial susceptibility testing (AST), and knowledge in the selection of antibiotics appropriate for organism and specimen type.

Expected Learning Outcomes:

By the end of this session, participants will be able to:

- Describe the importance of AST in AMR detection for diagnosis and surveillance.
- Describe techniques used for AST.
- Describe the rationale for use of a 0.5 McFarland Turbidity Standard.
- List the common classes of antimicrobial agents.
- Describe process for selection of colonies for AST.
- Select the appropriate antibiotic disks based on CLSI guidelines (dependent on organism and sample types).
- Explain how results are interpreted and reported, including AST expert rules and Minimum Inhibitory Concentration (MIC) results.
- Describe test modifications for AST and break points.
- Describe the necessary methods for QC.

Content Outline:

- Importance of AST in AMR detection for diagnosis and surveillance.
- Techniques used for AST.
- Rational for use of a 0.5 McFarland Turbidity Standard.
- List the common classes of antimicrobial agents.
- Describe process for selection of colonies for AST.
- Selection of colonies for AST.
- Selection of antibiotic disks based on guidelines.
- Modifications for AST and break points.
- Interpreting and reporting results, including AST expert rules, MIC, and clinical break points.
- AST guidelines: Clinical and Laboratory Standards Institute (CLSI) guidelines.
- Methods for QC.

5.7 Molecular Testing for Bacterial Identification and AMR

Purpose:To equip participants with skills in interpretation, quality assurance of molecular diagnostic techniques, and how to choose the most appropriate molecular technique for their setting.

Expected Learning Outcomes:

By the end of this module, participants will be able to:

- Describe the basics of Molecular identification.
- Explain the difference between phenotypic and genotypic AMR diagnostics.
- Outline molecular targets for identification and detection of priority bacterial pathogens.
- Outline molecular targets for AMR detection in priority bacterial pathogens.
- List available molecular testing platforms for AMR.
- List factors to consider in selecting appropriate molecular diagnostic tests.
- Describe benefits and limitations of molecular diagnostic tests.
- Explain how to interpret molecular tests results.
- Describe QC approaches for molecular tests.

Content Outline:

- Basics of molecular diagnostics.
- Difference between phenotypic and genotypic diagnostics.
- Molecular advanced technologies including Conventional PCR, QPCR, DNA microarrays and DNA Sequencing, including Whole Genome Sequencing (WGS), Next Generation Sequencing (NGS), Loop-Mediated Isothermal Amplification (LAMP), and Metagenomic Analysis.
- Molecular targets for identification of priority bacterial pathogens.
- AMR molecular targets for priority bacterial pathogens (e.g., Extended Spectrum Beta Lactamase, mec genes).
- Available molecular diagnostics platforms for bacterial identification and AMR.
- Benefits and limitations of molecular diagnostic tests.
- Interpretation of molecular diagnostic tests.
- Molecular tests QC.

MODULE 6: SPECIMEN REFERRAL AND REPORTING

Purpose:

The purpose of this module is to equip participants with knowledge and skills for sample/isolates referral and reporting.

Target Audience: Laboratory practitioners, Clinicians, veterinarians, veterinary paraprofessionals and any other relevant stakeholder or officer.

Expected Learning Outcomes:

By the end of this module, participants will be able to:

- Describe the tiered sample referral system.
- Explain the goals of sample referral.
- Explain when to refer a sample or isolate to a testing laboratory or to a reference laboratory.
- Describe documents that are used in sample referral and reporting.
- Describe microbiological critical results and notifiable infectious diseases.
- Describe how to relay results in a timely and accurate manner.

Content Outline:

- The tiered sample referral system.
- Goals of sample referral.
- Referral of a sample or isolate to a testing laboratory or to a reference laboratory.
- Documents that are used in sample referral and reporting.
- Microbiological critical results and notifiable infectious diseases.
- Relaying results in a timely and accurate manner.

MODULE 7: QUALITY ASSURANCE IN THE CLINICAL MICROBIOLOGY LABORATORY

Purpose:

The purpose of this module is to provide participants with knowledge and skills for quality assurance programs.

Target Audience: Laboratory practitioners, clinicians, veterinarians, veterinary paraprofessionals and any other relevant stakeholder or officer.

Expected Learning Outcomes:

By the end of this module, participants will be able to:

- Define the following terms: Quality, Quality Management Systems (QMS) Quality Control (QC), Quality Assurance (QA), Internal Quality Control (IQC), External Quality Assurance (EQA), Quality Indicators (QI), and Continuous Quality Improvement (CQI).
- Differentiate between QC and EQA.
- State the components of a QA program.
- Describe implementation of a QC program in microbiology laboratories.
- Outline errors in microbiology tests.
- Describe corrective and preventive action.
- Identify and monitor quality indicators.
- Outline measures used to implement a continual quality improvement process.

Content Outline:

- Define QMS, QC, IQC, QA, EQA, QI, and CQI.
- Difference between QC and EQA.
- Components of a QA program.
- Implementing a QC program in microbiology laboratories:
    - ◊ Reagents
    - ◊ Media
    - ◊ Antibiotic susceptibility testing
- Errors in microbiological testing.
- Documentation: corrective and preventive actions.
- Identification and monitoring of quality indicators (e.g., turnaround time, rejection rates, rate of contamination).
- Implementation of a Continuous Quality Improvement process.

MODULE 8: AMR SURVEILLANCE MONITORING, EVALUATION, AND REPORTING

8.1 AMR Surveillance

Purpose: To equip personnel with knowledge and skills for AMR surveillance.

Target Audience: HRIOs, M&E officers, ICT officers, data managers, data clerks, laboratory practitioners, public health and veterinary epidemiologists, veterinarians in charge of data, County Health Information Management team, clinicians any other relevant stakeholder or officer.

Expected Learning Outcomes:

By the end of this module, participants will be able to:

- Define AMR surveillance.
- Explain the importance of AMR surveillance.
- Describe the components of AMR surveillance.
- Describe the challenges to effective AMR surveillance.
- Describe the Global Antimicrobial Resistance Surveillance System (GLASS).
- Content Outline:
- Objectives and importance of AMR surveillance.
- Components of an AMR surveillance system.
- Challenges of an effective AMR surveillance system.
- Understanding the Global Antimicrobial Resistance Surveillance System (GLASS).

8.2 Data and Information Management

Purpose: To equip personnel with knowledge and skills for AMR data and information management.

Expected Learning Outcomes:

By the end of this module, participants will be able to:

- Understand the importance of AMR data.
- Explain the terms used in data management.
- Describe the type of AMR data collected.
- Understand the variables used in AMR surveillance.
- Identify and illustrate how to use various data collection and reporting tools for AMR surveillance.
- Describe AMR surveillance data flow.
- Identify guidelines and procedures required for efficient collection, storage, and retrieval of data.
- Understand the ethics of data usage and management.

Content Outline:

- Importance of AMR data and information.
- Terms used in data management.
- AMR data types and variables: data required for AMR surveillance.
- Data collection and reporting tools used for AMR surveillance.
- How to use AMR data collection and reporting tools.
- AMR data flow.
- AMR data governance and protection.

8.3 AMR Data Analysis and Reporting

Purpose: To equip personnel with knowledge and skills for AMR data analysis, presentation, and reporting.

Expected Learning Outcomes:

By the end of this module, participants will be able to:

- Define data analysis.
- Explain the importance of AMR data analysis.
- Understand data extraction (mining) and cleaning.
- Identify the various analyses to be conducted on AMR data.
- Demonstrate various data presentation techniques for AMR surveillance and generation of antibiogram reports.
- Understand AMR data reporting and dissemination among various stakeholders.
- Understand data storage and archiving.

Content Outline:

- Importance of AMR data analysis.
- AMR the data extraction (mining) and cleaning process.
- Descriptive data analysis.
- Presentation and reporting of AMR data and generation of antibiogram reports.
- Channels for sharing and disseminating AMR reports among stakeholders.
- Understanding data storage and archiving.

8.4 Integrated AMR Surveillance, One Health Approach

Purpose: To equip personnel with knowledge on integrated AMR surveillance, and the need for data sharing between the animal and human health sectors.

Expected Learning Outcomes:

By the end of this module, participants will be able to:

- Describe and understand an integrated AMR surveillance program.
- Understand the need for AMR data sharing among the animal and human health sectors.
- Understand data flow among the animal and human health sectors for AMR surveillance.
- Understand tools, software, and platforms for data sharing.

Content Outline:

- Integrated AMR surveillance.
- Need for AMR surveillance data sharing between the animal and human health sectors.
- Data flow between the animal and human health sectors for AMR surveillance.
- Tools, software, and platforms for data sharing.

8.5 Utilization of AMR Surveillance Data

Purpose: To familiarize personnel with the importance of data in AMR program monitoring and decision making (data driven decision making).

Expected Learning Outcomes:

- By the end of this module, participants will be able to:
- Understand the importance of quality data and timely reporting for decision making.
- Utilization of AMR data at different level (health facility/Lab, regional and national level).
- Describe the importance of utilizing surveillance data in program review, planning, advocacy, policy development, and decision-making processes.
- Describe the challenges of effective utilization of AMR surveillance data.

Content Outline:

- Quality data and timely reporting for decision making.
- Utilization of AMR data at different level (health facility/Lab, regional and national level).
- Importance of utilizing surveillance data in program review, planning, advocacy, policy development, and decision-making processes.
- Challenges of using AMR surveillance data.

MODULE 9: PROCUREMENT AND SUPPLY CHAIN MANAGEMENT

Purpose:

The purpose of this module is to equip personnel with knowledge and skills for ensuring that all equipment and supplies for microbiological testing are planned for, procured, and monitored appropriately.

Target Audience: laboratory practitioners, Administrator, Procurement officers and any other relevant stakeholder or officer.

Expected Learning Outcomes:

By the end of this module, participants will be able to:

- Identify all equipment and supplies required for the optimal performance of a microbiology laboratory.
- Explain the goals of an effective supply chain management system.
- Describe supply chain management processes.
- Describe the importance of inventory management and tools.
- Describe how to monitor supply chains performance.
- Prepare a forecast for essential microbiology supplies.

Content Outline:

- Equipment and supplies required for the optimal performance of microbiology laboratory.
- The goals of an effective supply chain management.
- Supply chain management processes.
- Factors to consider in products selection.
- Products quantification/forecasting.
- Procurement Processes; Products ordering and distribution models.
- Inventory management; Importance, types and tools.
- Reception, storage and assessing stock levels.
- Monitoring supply chains performance.
- Essential microbiology products forecast exercise.

MODULE 10: EQUIPMENT MANAGEMENT

Purpose:

The purpose of this module is to equip participants with knowledge and skills on how to establish a program for managing equipment in a microbiology laboratory (e.g., user maintenance schedules, warranty, service contracts, service level agreements, placements, and calibrations), and disposal and retirement of old and outdated equipment.

Target Audience: Laboratory practitioners, veterinarian paraprofessionals, public health personnel, biomedical engineers any other relevant stakeholder or officer.

Expected Learning Outcome:

By the end of this module, participants will be able to:

- Describe an overview of a microbiology equipment management program.
- Explain the selection of and processes required before equipment is put in use.
- Provide a rationale for developing a preventive maintenance program for their laboratory.
- Describe the requirements for a preventive maintenance program for equipment.
- Explain the process of documentation.
- Explain how to retire old, outdated, or obsolete laboratory equipment.
- Describe an equipment contingency plan.

Content Outline:

- Overview of a microbiology equipment management program.
- Items to consider prior to purchasing equipment for a microbiology laboratory.
- Selection and acquisition of new equipment.
- Rationale for developing an equipment preventive maintenance program (e.g., service contracts, service level agreements, placements, and calibrations).
- Requirements for a preventive maintenance program for equipment (daily, weekly, monthly, and annually).
- Process of documentation.
- Retirement of old, outdated, or obsolete laboratory equipment.
- Equipment contingency plan.

MODULE 11: CLINICAL GUIDE

Purpose:

The purpose of this module is to equip participants with knowledge and skills on the role of microbiology testing for the clinical management of patients and the prudent use of antibiotics.

Target Audience: Clinicians, nurses, pharmacists, laboratory practitioners any other relevant stakeholder or officer.

Expected Learning Outcomes:

By the end of this module, participants will be able to:

- Classify commonly used antibiotics.
- Describe the mechanism of antibiotic action and clinical use of each class.
- Outline the mechanisms of antibiotic resistance.
- Explain the factors considered when selecting antibiotics.
- Describe the role of microbiology testing in clinical management and AMR surveillance.
- Interpret microbiology results and AST/Antibiogram generation.
- Utilize antimicrobials prudently in clinical practice.

Content Outline:

- Commonly used antibiotics.
- Mechanism of action and clinical use of each antibiotic class.
- Mechanisms of antimicrobial resistance.
- Factors to consider when selecting antibiotics.
- Role of microbiology testing in clinical management and AMR surveillance.
- Interpretation of microbiology results and AST/ Antibiogram generation.
- Clinical management of infections from different body systems.

MODULE 12: PRUDENT USE OF ANTIMICROBIALS IN VETERINARY PRACTICE

Purpose:

The purpose of this module is to equip personnel with knowledge on the prudent use of antimicrobials.

Target Audience: Veterinarians, veterinary paraprofessionals and any other relevant stakeholder or officer.

12.1 Use of Antimicrobials

Expected Learning Outcomes:

By the end of this module, participants will be able to:

- Classify commonly used antibiotics in veterinary practice.
- Describe the mechanism of action of each antibiotics class.
- Explain the selection and clinical use of each class of antibiotics.
- Outline possible side effects for the antibiotics.
- Describe the restrictions of using critical human drugs in food animals.
- Discuss empiric antibiotic therapy for common infections.
- Have knowledge of the withdrawal periods for commonly used antibiotics.
- Illustrate the use of microbiology testing aid diagnosis and interpretation of results.
- Discuss case studies on the clinical management of infections from different body systems.

Content Outline:

- Classify commonly used antibiotics in veterinary practice in Kenya.
- OIE list of antimicrobial agents of veterinary importance.
- Mechanism of action of each antibiotic class.
- Antibiotics side effects.
- Restrictions of using critical human drugs in food animals.
- Factors to be considered before using antibiotics in animals.
- Empiric antibiotic therapy for common infections.
- Antimicrobial stewardship: disease prevention and reducing the need to use antibiotics.
- Pharmacokinetics and pharmacodynamics for antibiotic classes.
- Withdrawal periods for commonly used antibiotics.
- Importance of microbiology testing in diagnosis and interpretation of results.
- Clinical management of infections from different body systems.

12.2 Policy and Legislation in Antimicrobial Use

Purpose: To equip participants with knowledge on policy and legislation guiding antimicrobial use in animals.

Expected Learning Outcomes:

By the end of this module, participants will be able to:

- Apply prudent use of antibiotics in animals.
- Apply code of practice to minimize and contain antimicrobial resistance (CAC/RCP 61-2005).
- Explain Kenya guidelines for prudent use of antimicrobials in animals.
- Describe the veterinary surgeons and veterinary paraprofessionals act (the veterinary medicines directorate), 2015.

Content Outline:

- Code of practice to minimize and contain antimicrobial resistance (CAC/RCP 61-2005)
- Roles and responsibilities in prudent use of antimicrobials in animals
- Veterinary surgeons and veterinary paraprofessionals act (the veterinary medicines directorate), 2015.

COURSE EVALUATION AND DELIVERY METHODS

Participant expectations:

- To be informed on the importance of antimicrobial stewardship
- To be refreshed on prudent use of antibiotics
- To be refreshed on existing and informed of new technologies
- To be refreshed on pre- and post-analytical processes

Knowledge and skills transfer evaluation:

- Participant's pre- and post-test
- Trainer feedback and course evaluation

Course delivery methods:

- Lectures and PowerPoint presentations
- Role playing
- Demonstrations and observations of procedure
- Practical sessions
- Trainer-moderated group work sessions
- Case studies
- Use of visual aids

RESOURCES/LOGISTICS REQUIREMENTS

- Trainer and trainee manual
- Training modules
- PowerPoint presentations
- Supplies for exercises and practical sessions
- Laboratory practical sessions worksheets
- Room for demonstration
- Reference materials such as journals, policies, surveillance strategies, AMR action plan, and other relevant materials
- Estimated number of trainees: 25
- Pre- and post-test materials
- Course evaluation materials

REFERENCES

1. Ministry of Health, Kenya. (June 2017). National Policy on Prevention and Containment of Antimicrobial Resistance.
2. World Health Organization. (27 February to 3 March, 2017). Joint External Evaluation of IHR Core Capacities of the Republic of Kenya: Mission report.
3. World Health Organization. (2015). Global Action Plan on Antimicrobial Resistance: World Health Organization.
4. Ministry of Health, (June 2017). National Action Plan on Prevention and Containment of Antimicrobial Resistance: 2017-2022.
5. Ministry of Health, Kenya. (July 2018). National Antimicrobial Resistance Surveillance Strategy 2018-2020: Surveillance of Antimicrobial Resistance Using Public Health Laboratory Based Sentinel Sites in Kenya.

COURSE ORGANIZATION

MODULE	SUB MODULE	ACTIVITY/METHOD	CONTENT	PROPOSED TIME
Module 1: Overview of AMR.	1.1	PowerPoint presentation and Discussions	Overview of AMR.	45 Minutes
	1.2	PowerPoint presentation and Discussions	Kenya National Policy and Action Plan on AMR.	45 Minutes
	1.3	PowerPoint presentation and Discussions	Overview of AMR Surveillance Strategy.	50 Minutes
	1.4	PowerPoint presentation and Discussions	Introduction to Diagnostic Stewardship.	45 Minutes
	1.5	PowerPoint presentation and Discussions	Introduction to Antimicrobial Stewardship.	30 Minutes
	1.6	PowerPoint presentation and Discussions	Introduction to Infection Prevention and Control.	30 Minutes
Module 2: Drivers of Antimicrobial Resistance at the Human-Animal-Environment Interface.	2.0	PowerPoint presentation and Discussions	Drivers of AMR at Human-Animal-Environment Interface.	45 Minutes
Module 3: Introduction to Biosafety and Biosecurity.	3.0	PowerPoint presentation and Discussions	Introduction and Overview of Biosafety and Biosecurity.	2 Hours
		Interactive sessions	Case Scenarios and Exercises.	30 Minutes
Module 4: Specimen Collection, Transport, Reception, and Storage.	4.1.	PowerPoint presentation and Discussions	Specimen Collection, Transport, Reception, and Storage	2 Hours 30 Minutes
	4.1	Interactive sessions	Specimen Collection.	30 Minutes
	4.2	Practical	Packaging and Transport.	30 Minutes
	4.3	Practical	Specimen Receipt.	
	4.4	Practical	Storage.	
	4.5	Discussions and Interactive sessions	Custody of Samples and Isolates.	15 Minutes
Module 5: Microbiological Procedures.	5.1	Basic Microscopy<br>PowerPoint presentation	Proper Use of a Microscope;<br>Routine Maintenance of a Microscope.	45 Minutes
		Practical	Care and Maintenance of a Microscope.	1 Hour
	5.2	PowerPoint presentation	Routine Microscopy Procedures: Wet Mount, Smear Preparation; Gram Stain: Principle Method, Interpretation, and Quality Control.	1 Hour
		Practical	Wet Mount Preparation,<br>Smear Preparation,<br>Gram Staining.	2 Hours
	5.3	PowerPoint presentation	Types of Culture Media; Culture Media: Preparation, Storage, and Quality Control.	1 Hour
		Practical	Culture Media Preparation.	3 Hours
	5.4	PowerPoint presentation	Specimen Processing.	2 Hours
		Practical	Processing of Clinical/Surveillance specimens.	4 Hours
	5.5	PowerPoint presentation	Culture Reading and Bacteria Identification.	2 Hours
		Practical		2 Days
	5.6	PowerPoint presentation	Antibiotic Susceptibility Testing.	1 Hr 50 Mins
		Practical	AST Practical Demonstration.	30 Minutes
	5.7	PowerPoint presentation and Discussion	Molecular Testing for Bacterial Identification.	30 Minutes

Module 6:<br>Specimen Referral and Reporting.	6.0	PowerPoint presentation and Discussion	Specimen Referral and Reporting.	45 Minutes
		Interactive session	Case Scenario of Sample Referral Models.	30 Minutes
Module 7:<br>Quality Assurance in the Clinical Microbiology Laboratory.	7.0	PowerPoint presentation and Discussion	Quality Assurance, Route cause analysis, preventive/ corrective action, CQI.	2 Hours
		Exercises and Discussions	Route cause analysis-Fish borne model, CAPA.	30minitues
Module 8:<br>AMR Surveillance Monitoring, Evaluation, and Reporting.	8.1	PowerPoint presentation and Interactive sessions	AMR Surveillance and Challenges to Effective AMR Surveillance.	1 Hour 30 Minutes
	8.2	PowerPoint presentation and Interactive sessions	Data and Information Management and Importance of Data and Information.	1 Hour 25 Minutes
		Practical sessions	Use of Data Collection and Reporting Tools.	35 Minutes
	8.3	PowerPoint presentation and Interactive sessions	Data Analysis and Reporting, Definitions and Principles.	1 Hour 30 Minutes
		Practical	Data Cleaning, Mining, and Analysis.	2 Hours
	8.4	PowerPoint presentations	Principle of the "One Health" Approach.	45 Minutes
		Interactive sessions	Tools/Platforms for Data Sharing, Data Flow in the Various Sectors.	30 Minutes
	8.5	PowerPoint presentations	Using Surveillance Data for Decision Making.	45 Minutes
		Interactive sessions	Decisions That Can be Influenced by Data, Challenges in Utilizing Data.	30 Minutes
Module 9:<br>Procurement and Supply Chain Management.	9	PowerPoint presentations	Procurement and Supply Chain Management.	30 Minutes
		Practical	Review of Procurement Tools and Documentation.	15 Minutes
Module 10:<br>Equipment Management.	10.0	PowerPoint presentation	Equipment Management.	1 Hour 30 Minutes
		Exercises and Discussions	Review of Equipment Logs, Service Contracts, and Service Level Agreements.	15 Minutes
		Practical	Maintenance of equipment and documentation.	20 Minutes
Module 11:<br>Clinical Guide.	11	PowerPoint presentation	Clinical Guide on the Role of Microbiology Testing in Clinical Management of Patients and Prudent Use of Antibiotics.	3 Hours
		Brainstorming session	Discussions and plenary.	30 Minutes
		Interactive sessions	Case Studies.	1 Hours
Module 12:<br>Prudent Use of Antimicrobials in Veterinary Practice.	12.1	PowerPoint presentation	Use of Antimicrobials.	2 Hours
		Brainstorming session	Discussions and Plenary.	30 Minutes
		Interactive sessions	Case Studies.	2 Hour
	12.2	PowerPoint presentation and Discussion	Policy and Legislation in Antimicrobial Use.	2 Hours

LIST OF CONTRIBUTORS

STAKEHOLDERS AND PARTNERS		
No.	NAME	AFFILIATION
1	Dr. Evelyn Wesangula	Ministry of Health
2	Dr. Allan Azegele	Ministry of Agriculture Livestock and Fisheries
3	Dr. David Mutonga	USAID Infectious Disease Detection and Surveillance- Kenya
4	Mamo Umuro	National Public Health Laboratory
5	Bernard Sande	Head of diagnostic clinical services
6	Susan Githii	National Public Health Laboratory
7	Caroline Mbogori	National Public Health Laboratory
8	Dr. Andrew Thaiyah	United States Agency for International Development
9	Dr. Willie K Sang	Kenya Medical Research Institute
10	Dr. Willy Mwangi Edwin	University of Nairobi
11	Sheilla Chebore	USAID Infectious Disease Detection and Surveillance- Kenya
12	Dr. Loice Achieng	University of Nairobi
13	Dr. Marybeth Maritim	University of Nairobi
14	Prof Gunturu Revathi	Aga Khan University Hospital
15	Dr. Gilbert Kirui	University of Nairobi- Africa One Health University Network (*AFROHUN*)
16	Ahatha Abdi Mohamud	Kenya Medical Training Centre
17	Alfred Gitau	Kenyatta National Hospital
18	Dr. Gellian Omondi	Kenya Medical Research Institute
19	Mungai D. Ndung'u	USAID Infectious Disease Detection and Surveillance
20	Dr. Jafred Kitaa	University of Nairobi
21	Dr. Abubakar Abdillah	Aga Khan University Hospital
22	Dr. Alice Kanyua	Nairobi Hospital
23	Dr. Lillian Musila	Kenya Medical Research Institute
24	Dr. Linus Ndegwa	Centre for Disease Control-Kenya

25	Dr. Mary Romona Ndanyi	Directorate of Veterinary Services
26	Dr. Naomi Kemunto Peter	Washington State University
27	Dr. Naphtali Mwanziki	Directorate of Veterinary Medicine
28	Dr. Stella Kiambi	Food and Agriculture Organization
29	Dr. Valarie Magutu	University of Nairobi
30	Geoffrey Olela	National Public Health Laboratory
31	Gerald Murage	Technical University of Kenya
32	Jedidah Kehara	National Public Health Laboratory
33	John Mwihia	National Public Health Laboratory
34	Joshua Odero	USAID Infectious Disease Detection and Surveillance- Kenya
35	Josiah Mwenda	USAID Infectious Disease Detection and Surveillance- Kenya
36	Lydia Mudenyo	National Public Health Laboratory
37	Dr. Mbaire Chuchu	USAID Infectious Disease Detection and Surveillance- Kenya
38	Nelson Akenga	National Public Health Laboratory
39	Rodgers Norman Ndemba	National Public Health Laboratory-ECHO
40	Peter Kinyanjui Gatheca	National Public Health Laboratory
41	Pole Lewa Said	University of Nairobi
42	Prof. Samuel Kariuki	Kenya Medical Research Institute
43	Prof. Lilly Bebora	University of Nairobi
44	Stephen Kibe Njoroge	Jomo Kenyatta University of Agriculture and Technology
45	Dr. Susan Atieno Amuti	Directorate of Veterinary Services
46	Kennedy Mbogo	Kiambu county
47	Fredrick Mutai	Trans Nzoia County
48	Joseph Kimani	Kilifi County
49	Winfred Githinji	Nyeri County
50	Danson Kausu	United States Agency for International Development

51	Geofrey Olela	National Public Health Laboratory
52	Benard Mutie	National Public Health Laboratory
53	Winfred Kisiu	Kenyatta National Hospital
54	Brilliant Imungu	Machakos County
55	Dr. Oscar Agoro	Nyeri County
56	Dennis Kinyoki	Program for Appropriate Technology in Health – Kenya
57	Daniel Wekesa	Trans Nzoia County
58	Annette Wachira	Program for Appropriate Technology in Health – Kenya
59	Jane Mumbua	Centre for Veterinary Laboratory
60	William Wambugu	Muranga County
61	James Kariuki	Nyeri County
62	Dr. Zaituni Mulaa	Trans Nzoia County
63	Dr. Nathan Arwa	Agakhan University Hospital
64	Collins Jaguga	USAID- *Medicines, Technologies, and Pharmaceutical Services* (MTaPs)
65	Joseph Mkolo	USAID- *Medicines, Technologies, and Pharmaceutical Services* (MTaPs)
66	Dr. Florence Kangweini	Muranga County
67	Dr. Dorothy Aywak	Kenyatta National Hospital
68	Dr. Ruth Omani	Washington State University, Kenya
69	Isaac Baya	National Public Health Laboratory
70	Ben Muthamia	Family Health International 360- Kenya
71	Dr. Norah Maore	Nairobi County
72	Dr. Sylvia Omulo	Washington States University
73	Michael Kahara	Ministry of Agriculture Livestock and Fisheries
74	Dr. Thumbi Mwangi	Washington State University, Kenya
75	Philip Leitore	Family Health International 360- Kenya
76	Dr. Peninah Munyua	U.S. Centre for Disease Prevention and Control, Kenya
77	Dr. Maurice Wakwababu	Ministry of Health
78	Erastus Maugu	Infectious Disease Detection and Surveillance- Kenya

79	Dr Anicet Dahourou	Infectious Disease Detection and Surveillance
80	Dr. Ochiawunma Ibe	Infectious Disease Detection and Surveillance
81	Dr. Olivia Vélez	Infectious Disease Detection and Surveillance
82	Ali Kwizera	Infectious Disease Detection and Surveillance
83	Eva Muchira	Infectious Disease Detection and Surveillance
84	Dr. Lekopien C. Argeo	Zoonotic Disease Unit
85	Dr. Josephat Maina Kimani	Zoonotic Disease Unit
86	Mathew Mutiiria	Zoonotic Disease Unit
87	Mutono Nyamai	Washington States University

This document was made possible with the support of USAID's
Infectious Disease Detection and Surveillance (IDDS) Project
